# Age‐dependent changes and biomarkers of aging in *Caenorhabditis elegans*

**DOI:** 10.1111/acel.12853

**Published:** 2019-02-08

**Authors:** Heehwa G. Son, Ozlem Altintas, Eun Ji E. Kim, Sujeong Kwon, Seung‐Jae V. Lee

**Affiliations:** ^1^ Department of Life Sciences Pohang University of Science and Technology Pohang South Korea; ^2^ School of Interdisciplinary Bioscience and Bioengineering Pohang University of Science and Technology Pohang South Korea; ^3^ Present address: Institute of Intensive Care Medicine University Hospital Zurich, University of Zurich Zurich Switzerland

## Abstract

*Caenorhabditis elegans* is an exceptionally valuable model for aging research because of many advantages, including its genetic tractability, short lifespan, and clear age‐dependent physiological changes. Aged *C. elegans* display a decline in their anatomical and functional features, including tissue integrity, motility, learning and memory, and immunity. *Caenorhabditis elegans* also exhibit many age‐associated changes in the expression of microRNAs and stress‐responsive genes and in RNA and protein quality control systems. Many of these age‐associated changes provide information on the health of the animals and serve as valuable biomarkers for aging research. Here, we review the age‐dependent changes in *C. elegans* and their utility as aging biomarkers indicative of the physiological status of aging.

## 1 INTRODUCTION

Living organisms undergo many degenerative and deleterious functional and structural changes with aging. In animals, these changes include reduced motor activity, overall metabolic rates, and cognitive function, as well as impaired digestive function, and increased DNA damage. Delaying or reversing these degenerative changes is critical because one of the important goals of aging research is to prolong the healthy longevity of humans. To achieve this goal, a key initial step is to dene and characterize age‐dependent physiological and molecular changes. However, it takes a very long time for most mammals to display such age‐dependent changes. Therefore, short‐lived organisms have been used as valuable models for aging research.

*Caenorhabditis elegans* is one of the outstanding model organisms used for aging research because of its very short lifespan and simple physiology. Moreover, experiments with *C. elegans* are free of ethical concerns. Many breakthrough discoveries in the eld of aging research have been made using *C. elegans* because of these advantages (Kenyon, 2010). To date, the measurement of lifespan is the most popular method used to study aging in *C. elegans*. However, this assay still takes a relatively long time because the lifespan of wild‐type *C. elegans* is approximately 3 weeks. Additionally, chronological age does not always reflect biological (functional and physiological) age; a cohort with the same chronological age undergoes biological changes at variable rates, especially in the later stages of life. Therefore, dening quantitative changes associated with physiological aging, referred to as biomarkers of aging, is crucial for expediting the development of potential anti‐aging therapies.

**Figure 1:**
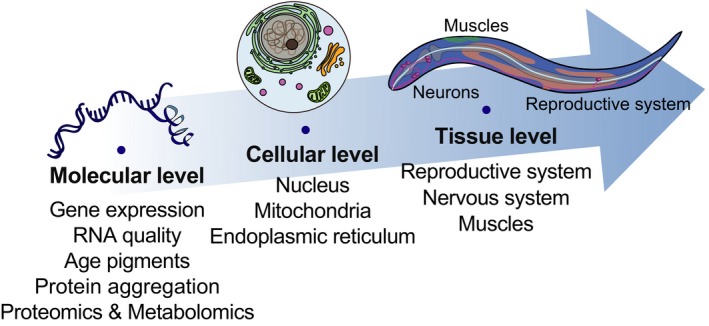
Age‐related changes in *Caenorhabditis elegans*. A schematic diagram shows age‐related changes that are discussed in this review. We classified age‐related changes into three levels: tissue, cellular, and molecular

Legitimate biomarkers of aging should exhibit changes in expression level or activity during physiological aging and therefore serve as a reference to predict the status of aging without harming the subjects. Thus, prominent age‐dependent molecular and physiological changes are reasonable candidates for biomarkers of aging. The characteristics of aging in *C. elegans* have been described in another review article (Collins, Huang, Hughes, & Kornfeld, 2008). In addition, a recently published book chapter is an outstanding resource for the age‐dependent anatomical changes of *C. elegans* (Herdon, Wolkow, Driscoll, & Hall, 2017). Here, we summarize the recent ndings regarding potential biomarkers of aging in *C. elegans*. We classify and describe age‐related changes in *C. elegans* at the tissue, cellular, and molecular levels (Figure 1). We also review the roles of insulin/IGF‐1 signaling (IIS) and dietary restriction (DR), two evolutionarily conserved aging‐modulating regimes, in age‐related changes. We then discuss the potential utility of these changes as biomarkers of aging in *C. elegans*. We believe that these biomarkers will provide valuable insights into the aging process and that this information will advance our understanding of aging and age‐related diseases in mammals, including humans.

## 2 AGE‐DEPENDENT CHANGES

### 2.1 Age‐dependent changes at the tissue level

#### 2.1.1 Reproductive system

**Figure 2:**
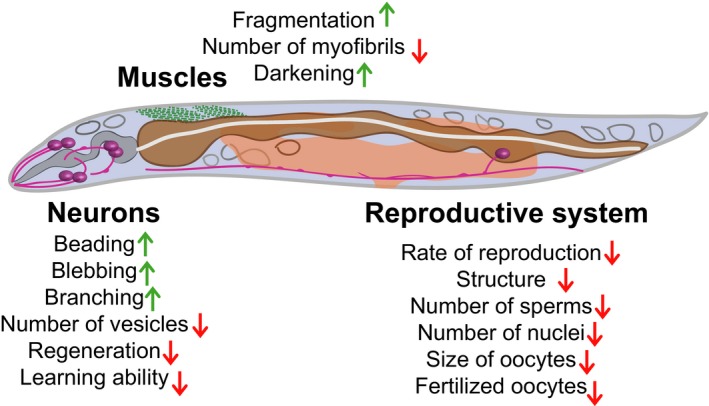
Age‐related changes at the tissue level. As *Caenorhabditis elegans *ages, the integrity of muscles, neurons, and the reproductive system declines. Muscles become fragmented, and the tissues of the reproductive system degenerate. Neuronal integrity appears to be prolonged compared to other tissues, but neurons also undergo age‐dependent deterioration, such as blebbing

Like many other physiological systems, the reproductive system of *C. elegans* undergoes age‐dependent changes (Figure 2). The rate of reproduction signicantly decreases with age, and the structure of the reproductive system deteriorates (Garigan et al., 2002; Hughes, Evason, Xiong, & Kornfeld, 2007; Luo, Kleemann, Ashraf, Shaw, & Murphy, 2010; McGee, Day, Graham, & Melov, 2012; Pickett, Dietrich, Chen, Xiong, & Kornfeld, 2013). As *C. elegans* is a self‐fertile hermaphrodite with a limited number of sperm (Ward & Carrel, 1979), sperm shortage is one of the reasons for reduced progeny at an early age. This is because the number of progeny produced by unmated (self‐fertilized) *C. elegans* decreases sharply and relatively early (Day 3) in adulthood (Hughes et al., 2007). In contrast, age‐dependent reductions in the number of progeny are delayed in mated hermaphrodites that receive sperm from other males compared to unmated hermaphrodites (Hughes et al., 2007; Luo et al., 2010). However, mated hermaphrodites also cease reproduction after a certain time point, implying the existence of factors, other than sperm shortage, that limit the reproductive period. Age‐associated deterioration of tissues in the reproductive system may hamper prolonged reproduction, despite the supply of extra sperm at an advanced age. In aged worms, the number of nuclei in the mitotic germline diminishes and the nucleoplasm displays an increased accumulation of grainy material and cavities (Garigan et al., 2002). Oocyte size also decreases during aging, which correlates with their reduced quality, suggesting that an appropriate oocyte size is crucial for reproduction (Andux & Ellis, 2008). In addition, the number of unfertilized oocytes in mated hermaphrodites increases with age (Luo et al., 2010). Thus, the germline of old worms undergoes sperm shortages and structural changes, which eventually render the worms sterile.

Mechanisms of reduced reproductive potency in aged male *C. elegans* have also been proposed (Chatterjee et al., 2013; Guo, Navetta, Gualberto, & Garcia, 2012). Although the activity, morphology, and number of sperm are preserved during the rst 3 days of adulthood, 3‐day‐old adults exhibit uncoordinated mating behaviors and a rapid decline in reproductive potency (Chatterjee et al., 2013; Guo et al., 2012). This uncoordinated mating behavior appears to be caused by hyperexcited muscles in the male reproductive system (Guo et al., 2012), as decreasing the activity of these hyperexcited muscles has been shown to improve mating potency. Overall, the reproductive systems of both hermaphrodites and males display age‐related changes, which lead to a reduction in the reproductive potential.

#### 2.1.2 Nervous system

Although it was initially reported that *C. elegans* neurons remain relatively intact during aging compared with other tissues (Collins et al., 2008; Herndon et al., 2002), subsequent studies have shown that neurons display subtle but reproducible age‐dependent changes (Chen, Chen, Jiang, Chen, & Pan, 2013; Chew, Fan, Gotz, & Nicholas, 2013; Collins et al., 2008; Herndon et al., 2002; Pan, Peng, Chen, & McIntire, 2011; Tank, Rodgers, & Kenyon, 2011; Toth et al., 2012; Figure 1). Touch receptor neurons, such as ALM neurons, display gradual beading, blebbing, and branching during aging (Pan et al., 2011; Tank et al., 2011). In addition, the number of vesicles per synapse in touch receptor neurons is signicantly lower in aged worms than in young worms (Toth et al., 2012). This indicates that the synaptic integrity of worms also degenerates during aging. The extent of aberrant neuronal changes varies among worms of the same age, and neuron integrity shows a positive correlation with the coordinated motility of worms. In addition to the touch receptor neurons, GABAergic motor neurons in aged worms display neurite branching (Tank et al., 2011). Another study, however, suggested that GABAergic motor neurons undergo minimal age‐related changes (Toth et al., 2012). Further studies are needed to resolve these discrepancies, which perhaps originate from dierences in experimental conditions.

In addition to these structural changes, aged *C. elegans* display a functional deterioration of neurons (Hammarlund, Nix, Hauth, Jorgensen, & Bastiani, 2009; Kauman, Ashraf, Corces‐Zimmerman, Landis, & Murphy, 2010; Liu et al., 2013; Murakami & Murakami, 2005). *Caenorhabditis elegans* motor neurons undergo functional declines starting at an early age (Liu et al., 2013) and display reduced regeneration capacity (Hammarlund et al., 2009; Kauman et al., 2010; Liu et al., 2013; Murakami & Murakami, 2005). Associative learning ability also decreases with age (Hammarlund et al., 2009; Kauman et al., 2010; Murakami & Murakami, 2005). Thus, dierent neuron types undergo dierent age‐dependent structural and functional declines.

#### 2.1.3 Muscles

An age‐dependent reduction in the muscle integrity of *C. elegans* has been widely reported (Chow, Glenn, Johnston, Goldberg, & Wolkow, 2006; Garigan et al., 2002; Glenn et al., 2004; Herndon et al., 2002; Johnston, Iser, Chow, Goldberg, & Wolkow, 2008; Shamir, Wolkow, & Goldberg, 2009; Figure 1). Nuclei and sarcomeres in the body wall muscle cells undergo age‐dependent changes. For example, nuclei in the body wall muscle cells undergo redistribution with age. However, the extent of this redistribution varies between as well as within individual worms, suggesting that stochastic factors play a role in this phenomenon (Herndon et al., 2002). Additionally, the size of muscle cell nucleoli tends to increase during aging (Herndon et al., 2002, see also Tiku et al., 2017). Furthermore, sarcomeres in the body wall muscles lose their densely packed structures and regular orientations with increasing age (Glenn et al., 2004; Herndon et al., 2002). The *C. elegans* pharynx, a neuromuscular organ composed of 20 muscle cells and 20 neurons, also loses its integrity during aging (Albertson & Thomson, 1976; Chow et al., 2006; Garigan et al., 2002; Herndon et al., 2002; Johnston et al., 2008; Podshivalova, Kerr, & Kenyon, 2017; Zhao et al., 2017). In aged worms, the number of myobrils diminishes and the pharyngeal muscles exhibit an abnormal appearance (Chow et al., 2006; Garigan et al., 2002; Herndon et al., 2002; Podshivalova et al., 2017; Zhao et al., 2017). Other age‐dependent degenerative structural changes, such as darkening of the pharyngeal regions, also occur (Chow et al., 2006; Garigan et al., 2002; Herndon et al., 2002). These age‐dependent functional and structural abnormalities of pharyngeal muscles appear to be associated with bacterial accumulation in the terminal bulb of old worms (Podshivalova et al., 2017; Zhao et al., 2017). This is consistent with the susceptibility of old worms to infection by pathogenic bacteria, including *Pseudomonas aeruginosa* (Youngman, Rogers, & Kim, 2011). Degenerative changes in the muscle morphology of *C. elegans* have been conrmed via quantitative analytical methods (Johnston et al., 2008; Shamir et al., 2009). Thus, the loss of muscular integrity in old *C. elegans* results in age‐related changes, including impaired motility and increased susceptibility to infection, as in humans.

### 2.2 Age‐dependent changes at the cellular level

#### 2.2.1 Nucleus

**Figure 3:**
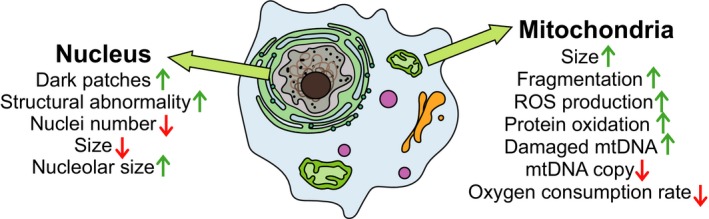
Age‐related changes at the cellular level. A schematic diagram that shows changes in the nuclei and mitochondria of old *Caenorhabditis elegans* cells

The integrity of *C. elegans* nuclei generally diminishes with age, although the degree of deterioration may vary among tissues (Garigan et al., 2002; Golden et al., 2007; Haithcock et al., 2005; Herndon et al., 2002; McGee et al., 2011; Figure 3). The intestine of adult *C. elegans* has approximately 30–34 cells whose nuclei start to degenerate at Day 12 of adulthood, resulting in a reduction in their number and size (McGee et al., 2011). The nuclei of muscle cells also develop dark patches with age (Haithcock et al., 2005; Herndon et al., 2002), and the relative size of the nucleoli increases with aging (Herndon et al., 2002; Tiku et al., 2017). Likewise, hypodermal and pharyngeal cell nuclei exhibit age‐associated abnormalities (Haithcock et al., 2005). The nuclear lamina, which supports the structure of nucleus, displays irregular peripheral structures in the body wall muscle, hypodermal, and intestinal cells (Haithcock et al., 2005). The space between the nuclei of syncytial germ cells increases, and the number of the nuclei decreases during aging (Garigan et al., 2002). Overall, nuclei in *C. elegans* undergo age‐dependent structural changes to varying degrees.

#### 2.2.2 Mitochondria

Mitochondria undergo age‐dependent structural and functional changes as well (Gruber et al., 2011; Yasuda et al., 2006; Figure 3). For example, aged mitochondria in the body wall muscle cells become enlarged and swollen. These changes seem to be caused by mitochondrial fusion rather than an increase in the size of individual mitochondria because the total mitochondrial mass does not change during aging (Yasuda et al., 2006). Old worms also display fragmented mitochondria (Hahm, Kim, DiLoreto, & Shi, 2015; Regmi, Rolland, & Conradt, 2014; Roux, Langhans, Huynh, & Kenyon, 2016), and this correlates with uncoordinated locomotion in aged *C. elegans* (Hahm et al., 2015). Mitochondria are the major organelles that produce reactive oxygen species (ROS) that oxidize proteins and induce structural DNA damage, thus impairing the function of mitochondria (Birben, Sahiner, Sackesen, Erzurum, & Kalayci, 2012). Increases in the level of ROS, carbonylated proteins, and damaged mitochondrial DNA have been reported during aging in *C. elegans* (Berlett & Stadtman, 1997; Cui, Kong, & Zhang, 2012; Gruber et al., 2011; Stadtman & Berlett, 1997; Yasuda et al., 2006). Consistent with these changes, mitochondrial DNA copy numbers (Gruber et al., 2011) and oxygen consumption rates (Gruber et al., 2011; Yasuda et al., 2006) decrease during aging. Thus, age‐dependent declines in the integrity of mitochondria appear to be associated with functional impairments.

#### 2.2.3 Endoplasmic reticulum

The endoplasmic reticulum (ER) is an organelle where proteins and lipids are synthesized and modied and serves as a calcium reservoir. The ER is also a site where the unfolded protein response (UPR) or ER^UPR^ occurs (Smith & Wilkinson, 2017). When misfolded proteins accumulate under various stress conditions, the ER^UPR^ is activated to degrade the misfolded proteins through three key pathways including the protein kinase RNA‐like ER kinase (PERK), the inositol‐requiring enzyme‐1 (IRE‐1)/X‐box binding protein‐1 (XBP‐1), and the activating transcription factor 6 (ATF6) pathways. The capacity of the ER^UPR^ seems to be reduced during aging, as has been determined by measuring the levels of HSP‐4, a target chaperone of IRE‐1/XBP‐1, under ER stress conditions (Ben‐Zvi, Miller, & Morimoto, 2009; Maity et al., 2016; Martinez, Duran‐Aniotz, Cabral‐Miranda, Vivar, & Hetz, 2017; Taylor & Dillin, 2013). In addition, the overexpression of XBP‐1 in neurons is sucient for enhancing ER stress resistance and longevity (Taylor & Dillin, 2013). Overall, these results suggest that the ER^UPR^ plays a functional role in aging and lifespan.

### 2.3 Age‐dependent changes at the molecular level

#### 2.3.1 Gene expression

It is not surprising that the expression of many genes is altered during *C. elegans* aging (Cortes‐Lopez et al., 2018; de Lencastre et al., 2010; Golden, Hubbard, Dando, Herren, & Melov, 2008; Golden & Melov, 2004; Kato, Chen, Inukai, Zhao, & Slack, 2011; Lund et al., 2002; Rangaraju et al., 2015; Vinuela, Snoek, Riksen, & Kammenga, 2012; Youngman et al., 2011). For example, mRNA levels of heat‐shock protein‐encoding genes increase until reaching midlife and then decrease in old age (Golden & Melov, 2004; Golden et al., 2008; Lund et al., 2002). In addition, the expression of many collagen genes, whose overexpression increases lifespan, decreases with age (Ewald, Landis, Porter Abate, Murphy, & Blackwell, 2015; Golden et al., 2008). Many other aging‐associated gene expression changes have been identied, but we still do not fully understand the eects of global gene expression changes on aging. It will be important to determine whether these gene expression changes have causative roles in aging or are the consequences of aging.

The expression of many noncoding RNAs, including microRNAs (miRNAs), Piwi‐interacting RNAs (piRNAs), transfer RNAs (tRNAs), ribosomal RNAs (rRNAs), small nucleolar RNAs (snoRNAs), and circular RNAs (circRNAs), changes with age (Cortes‐Lopez et al., 2018; de Lencastre et al., 2010; Ibanez‐Ventoso et al., 2006; Inukai & Slack, 2013; Kato & Slack, 2013; Kato et al., 2011; Lucanic et al., 2013). The overall levels of miRNAs tend to decrease during aging (de Lencastre et al., 2010; Ibanez‐Ventoso et al., 2006), and this appears to be caused by age‐dependent reductions in the mRNA levels of *dcr‐1*/Dicer (Mori et al., 2012) and *alg‐1*/Argonaut (Inukai, Pincus, de Lencastre, & Slack, 2018). The mRNA levels of *alg‐1* appear to be decreased by miR‐71, whose levels increase with age (de Lencastre et al., 2010; Inukai et al., 2018). Interestingly, miR‐71 is necessary and sucient for longevity (de Lencastre et al., 2010), suggesting a role for miR‐71 in aging through its regulation of *alg‐1*. The overall levels of tRNAs, rRNAs, and snoRNAs increase with age, whereas the levels of piRNAs decrease with age (Kato et al., 2011). circRNAs are a recently identied type of noncoding RNAs, whose ends are covalently linked and are implicated in transcriptional regulation (Knupp & Miura, 2018). A recent study indicated that the overall levels of circRNAs increase during *C. elegans* aging (Cortes‐Lopez et al., 2018). Whether various noncoding RNAs, other than miRNAs, act as biomarkers of aging and/or aect lifespan remains to be determined.

#### 2.3.2 RNA quality

RNA quality control mechanisms decline during aging in *C. elegans* (Heintz et al., 2017; Son et al., 2017). Nonsense‐mediated mRNA decay (NMD) is a cellular protective pathway that degrades mRNAs containing premature termination codons (PTCs), as these generate potentially toxic truncated proteins (Miller & Pearce, 2014). NMD activity decreases during aging in the tissues of various organs, including the muscles, hypodermis, and intestine (Son et al., 2017). In addition, mRNA splicing delity decreases with age, as shown by the increased levels of introns and unannotated regions in the mRNAs of aged worms (Heintz et al., 2017). Age‐dependent declines in RNA quality control negatively aect longevity, because enhancing NMD activity or overexpressing key splicing factors increases lifespan (Heintz et al., 2017; Son et al., 2017).

mRNAs that are destined to be degraded, such as those targeted by miRNAs, are stored and degraded in processing bodies (P‐bodies; Chantarachot & Bailey‐Serres, 2018). In the P‐body, most mRNAs are degraded by decapping enzymes and exonucleases. DCP1/decapping protein 1 and DCP2/decapping protein 2 remove the 5' cap of target mRNAs, and then the XRN1/5'‐3' exoribonuclease fully degrades the mRNAs (Labno, Tomecki, & Dziembowski, 2016). Interestingly, the inhibition of RNA decay by RNAi targeting *xrn‐1*/XRN1 increases the number of *dcp‐1::gfp* granules, which are also increased during aging (Rousakis et al., 2014). These results suggest that RNA decay eciency decreases during aging. Furthermore, perturbation of the RNA decay components aects lifespan; genetic inhibition of *dcap‐1*/DCP1, *dcap‐2*/DCP2, or *xrn‐1*/XRN1 decreases lifespan in *C. elegans* (Rousakis et al., 2014). In contrast to P‐body granules, aging does not aect the number of stress granules that are induced by various stresses (Chantarachot & Bailey‐Serres, 2018; Rousakis et al., 2014). Overall, the capacity for mRNA decay and proper mRNA metabolism decreases with age.

#### 2.3.3 Age pigments

Organisms contain various types of fluorescent materials, including aromatic amino acids, lipofuscin, advanced glycation end products, and anthranilic acids, several of which accumulate with age. Lipofuscin is well known as an “age pigment,” as its level increases with aging in many organisms (Gray & Woulfe, 2005; Klass, 1977; Xu, Chen, Manivannan, Lois, & Forrester, 2008). Age pigments can be quantitated using in vivo uorescence spectroscopy that detects a broad range of excitation/emission spectra. The accumulation of age pigments is gradual until early adulthood (Days 5–10) and rapid in midlife (Days 10–15; Gerstbrein, Stamatas, Kollias, & Driscoll, 2005). Furthermore, the absolute amount of generated age pigments negatively correlates with health and lifespan, and possibly predicts the lifespan of *C. elegans* (Gerstbrein et al., 2005; Pincus, Smith‐Vikos, & Slack, 2011). However, a study by the Gems group challenges the view of lipofuscin as an age pigment in *C. elegans* (Coburn et al., 2013). They have shown that kynurenines, which emit blue uorescence, exhibit characteristics similar to those of lipofuscin. Kynurenine levels do not increase gradually with aging, but they are precipitously elevated upon the death of the organism, resulting in sudden bursts of blue uorescence (Coburn et al., 2013). A recent study proposes that substances emitting red signals increase gradually with age (Pincus, Mazer, & Slack, 2016), whereas those emitting blue signals increase only marginally during aging and dramatically after death (Coburn et al., 2013; Pincus et al., 2016). Further characterization of the chemical nature of age pigments is required to resolve this issue.

#### 2.3.4 Protein aggregation

Protein homeostasis generally declines during aging, and this decline is associated with age‐related diseases. For example, aggregated proteins contribute to the pathology of neurodegenerative diseases, such as Alzheimer's, Parkinson's, and polyglutamine diseases in humans (Nollen et al., 2004; Taylor, Hardy, & Fischbeck, 2002). *C. elegans* displays an aging‐associated aggregation of transgene‐encoded polyQ and α‐synuclein, which are models for Huntington's and Parkinson's diseases, respectively (Morley, Brignull, Weyers, & Morimoto, 2002; van Ham et al., 2008). Many inherent proteins become insoluble and form aggregates in aged worms (David et al., 2010; Reis‐Rodrigues et al., 2012; Walther et al., 2015). Genetically inhibiting each of many aggregation‐prone protein‐coding genes increases lifespan (Reis‐Rodrigues et al., 2012), suggesting that protein aggregation has a negative eect on longevity. Thus, aging processes are accompanied by an increase in the accumulation of nonfunctional and insoluble protein aggregates.

#### 2.3.5 Proteomic and metabolomic changes

“Omics” techniques allow researchers to examine universal changes in genes, RNAs, proteins, and metabolites during aging. Using stable isotope labeling with amino acids in cell culture (SILAC) followed by LC‐MS/MS methods, *C. elegans* proteins whose levels increase or decrease with age have been reported (Narayan et al., 2016). During aging, proteins that likely function in nucleosome assembly, ER‐nuclear signaling, and the response to unfolded proteins increase, whereas the abundance of proteins involved in metabolism such as fatty acid, carbohydrate, and amino acid metabolism decreases (Narayan et al., 2016). A similar observation has been reported showing that overall levels of fatty acid metabolic proteins decrease (Copes et al., 2015). Because altering fat metabolism has huge impacts on aging and lifespan (Lee et al., 2015; Lemieux & Ashra, 2016), the proper maintenance of fat metabolism in old age will be benecial for longevity.

Levels of metabolites such as amino acids change with age as well. A study using ultra performance liquid chromatography (UPLC)‐MS/MS indicated that the levels of all of the amino acids except glycine and aspartic acid increase at an early age (approximately 3 days of adulthood) and then decrease sharply afterward (Gao et al., 2017). Another study using proton NMR spectroscopy reported that the levels of three amino acids (glycine, serine, and tyrosine) increase during aging, whereas the levels of four amino acids (alanine, asparagine, glutamate, and glutamine) decrease during aging (Davies, Bundy, & Leroi, 2015). Importantly, some of these age‐dependent changes reflect the biological age of worms (Davies et al., 2015), and treatment with any amino acids except phenylalanine and aspartate increases lifespan (Edwards et al., 2015). Further studies are required to comprehensively understand the roles of age‐dependent changes in amino acid levels in aging and longevity.

## 3 THE ROLE OF INSULIN/IGF‐1 SIGNALING AND DIETARY RESTRICTION IN AGE‐ASSOCIATED CHANGES IN *C. elegans*

Many intrinsic and extrinsic lifespan regulatory factors, including insulin/IGF‐1 signaling (IIS), target of rapamycin (TOR), mitochondrial respiration, reproduction, and dietary restriction (DR), have been identied using *C. elegans*. Among them, here we review age‐related changes under reduced IIS and DR conditions, which are two representative regimens for extending lifespan and delaying aging. The IIS pathway is an evolutionarily conserved aging regulatory pathway (Altintas, Park, & Lee, 2016; Kenyon, 2010). Mutations in the *daf‐2*/insulin/IGF‐1 receptor double the lifespan of *C. elegans* by upregulating various transcription factors, including DAF‐16/FOXO, heat‐shock factor 1 (HSF‐1), and SKN‐1/NRF. DR also promotes longevity across phyla (Kapahi, Kaeberlein, & Hansen, 2017). *Caenorhabditis elegans eat‐2* mutants, which display reduced feeding rates and food intake, are a widely used DR model (Lakowski & Hekimi, 1998; Raizen, Lee, & Avery, 1995). Reduced IIS and DR delay various age‐related degenerative changes. The motility of worms, as measured by maximum velocity, is prolonged in *daf‐2* and *eat‐2* mutants compared to wild‐type animals (Hahm et al., 2015; Huang, Xiong, & Kornfeld, 2004), although *daf‐2* mutants display reduced spontaneous movements for a relatively long time (Bansal, Zhu, Yen, & Tissenbaum, 2015). Additionally, *daf‐2* mutants are resistant to bacterial colonization (Podshivalova et al., 2017), one of the main causes of early death in *C. elegans* (Garigan et al., 2002; Zhao et al., 2017). Moreover, the reproductive span of *daf‐2* and *eat‐2* mutant hermaphrodites (Hughes et al., 2007; Luo et al., 2010; Luo, Shaw, Ashraf, & Murphy, 2009) is longer than that of wild‐type worms, although the total number of progeny produced by *daf‐2* mutants is reduced (Hughes et al., 2007). Mutations in *daf‐2* also prolong the reproductive period of males (Chatterjee et al., 2013). Furthermore, *daf‐2* mutants exhibit reduced uterine mass (McGee et al., 2012) and delayed deterioration of germ cells (Garigan et al., 2002) compared to wild‐type worms. Age‐dependent increases in neuronal abnormalities, including neurite branching and irregularly shaped soma in mechanosensory neurons, are delayed in *daf‐2* mutants compared to the wild type (Pan et al., 2011; Tank et al., 2011). Axon regeneration capacity is also prolonged in *daf‐2* mutants compared to the wild‐type animals (Byrne et al., 2014). In contrast, neither neurite branching nor axon regeneration appears to be delayed by mutations in *eat‐2* (Byrne et al., 2014; Tank et al., 2011). Neuronal functions, such as associative learning ability, are maintained longer in both *daf‐2* and *eat‐2* mutants than in wild‐type worms (Kauman et al., 2010).

Nuclear integrity in diverse tissues is maintained for a longer period of time in *daf‐2* mutants compared to in wild‐type worms, as exemplied by the delayed degeneration of *daf‐2* mutant nuclear lamina (Haithcock et al., 2005). Mutations in *age‐1*, the gene that encodes the phosphoinositide 3‐kinase acting immediately downstream of DAF‐2, delay nuclear degeneration in muscle (Herndon et al., 2002). In addition, the age‐related loss of intestinal nuclei (McGee et al., 2011) and hypodermal nuclei (Golden et al., 2007) is reduced in *daf‐2* mutants. Nucleolar size is negatively correlated with longevity and is reduced in *daf‐2* and *eat‐2* mutants (Tiku et al., 2017). In addition, *daf‐2* mutants display higher ATP levels and antioxidant activity and lower ROS levels than wild‐type worms (Zarse et al., 2012). This indicates that mitochondrial activity is increased, while ROS levels are reduced, likely because of an upregulation of antioxidants in *daf‐2* mutants (Zarse et al., 2012). RNAi knockdown of *daf‐2* during adulthood, however, transiently increases the level of ROS, which acts as a signal for longevity (Zarse et al., 2012), suggesting a stage‐specic regulation of ROS levels by IIS.

Mutations in *daf‐2* delay the age‐associated accumulation of abnormal macromolecules, including mRNAs with PTCs (Son et al., 2017) and insoluble proteins (David et al., 2010). Age pigment accumulation is reduced both in *daf‐2* mutants and in *eat‐2* mutants (Gerstbrein et al., 2005). Overall, the delay in age‐related degenerative changes caused by *daf‐2* mutations and DR is consistent with the idea that reduced IIS and DR confer healthy aging with a long lifespan.

## 4 POTENTIAL BIOMARKERS OF AGING

Biomarkers of aging reflect the physiological and functional age of organisms (Baker & Sprott, 1988). An important way to validate biomarkers of aging is by testing whether they predict the remaining lifespan of an organism (Baker & Sprott, 1988; Johnson, 2006). Several biomarkers predictive of lifespan have been reported in *C. elegans* (Table 1). First, physiological functions are indicators of the biological age of worms. For example, worms that display fast locomotion during early adulthood (Hahm et al., 2015; Huang et al., 2004; Pincus et al., 2011) or maintain their youth speed in middle age (Hsu, Feng, Hsieh, & Xu, 2009) tend to have longer lifespans compared with slow‐moving worms. The rate of pharyngeal pumping and the number of progeny after mating also positively correlate with remaining lifespan (Huang et al., 2004; Pickett et al., 2013). Second, nucleolar size has a negative correlation with longevity (Tiku et al., 2017). Importantly, nucleolar size is reduced by interventions that extend lifespan in *Drosophila melanogaster*, mice, and possibly humans as well (Tiku et al., 2017). Third, molecular genetic changes are used as biomarkers of aging. The expression levels of several genes, including a small heat‐shock protein (*hsp‐16.2*) after transient heat shock (Rea, Wu, Cypser, Vaupel, & Johnson, 2005) and superoxide dismutase 3 (*sod‐3;* Sanchez‐Blanco & Kim, 2011), are positively correlated with a long lifespan. In addition, the expression levels of certain miRNAs increase or decrease as worms grow old. Changes in these miRNA levels successfully predict the remaining lifespan of *C. elegans* (Pincus et al., 2011). Fourth, the amount of autofluorescent age pigments has been used as biomarkers of aging. These age pigments accumulate in old worms, and worms with a high accumulation of age pigments early in adulthood tend to live short lives (Pincus et al., 2016, 2011 ). Finally, mitochondrial functional integrity also predicts lifespan. Superoxide bursts (Cheng et al., 2014; Shen et al., 2014) or pH changes (Schwarzlander et al., 2014) in the mitochondria, referred to as “mitoashes,” early in adulthood (Day 3) show a negative correlation with lifespan. However, morphological changes of the mitochondria do not correlate with lifespan (Regmi et al., 2014) and therefore do not seem to be suitable as a biomarker of aging. Overall, physiological and molecular factors, age pigments, and mitochondrial integrity parameters are important biomarkers of aging in *C. elegans*. Whether these biomarkers are applicable to other organisms needs to be validated. After validation, these universal biomarkers of aging will be very helpful in understanding the mechanistic and physiological causes and consequences of the aging process in multiple species, including mammals.

**Table 1:** List of potential biomarkers of aging in *Caenorhabditis elegans*

	Biomarkers of aging	Correlation with lifespan	References
Physiological markers	Locomotion	Positive	Hahm et al. (2015), Hsu et al. (2009), Huang et al. (2004) and Pincus et al. (2011)
	Pharyngeal pumping rate	Positive	Huang et al. (2004)
	Progeny number	Positive	Pickett et al. (2013)
Cellular markers	Nucleolar size	Negative	Tiku et al. (2017)
Molecular markers	*sod* *–3* expression	Positive	Sanchez‐Blanco and Kim (2011)
	*hsp* *–16* *.2* expression	Positive	Rea et al. (2005)
	miRNAs	Positive or negative	Pincus et al. (2011)
	Age pigments	Negative	Pincus et al. (2011) and Pincus et al. (2016)
	Mitochondrial activity	Negative	Cheng et al. (2014) and Shen et al. (2014)

## 5 CONCLUSIONS

Risks for many chronic diseases, including cancer, cardiovascular diseases, and neurodegenerative diseases, exponentially increase with age. Research aiming at understanding the mechanisms of aging or delaying aging can be benecial for reducing these risks. The development of biomarkers of aging is crucial for aging research, as they reflect the biological age of an organism. In this review, we described age‐related changes that can potentially be used as biomarkers of aging in *C. elegans*.

So far, diverse, promising biomarkers of aging have been identied in *C. elegans*. Several limitations, however, need to be addressed in future research. First, the validity of several potential biomarkers of aging is controversial, and, therefore, further studies resolving these discrepancies are needed. Second, a biomarker of aging that reports one physiological or physical state usually does not reflect the general biological age of an organism. For example, a single worm that displays increased overall motility may have a reduced feeding rate. Therefore, it will be important to use multiple biomarkers of aging collectively, from physiological markers to molecular markers, to precisely estimate the biological age of an organism. In this sense, the standardization of which combinations of biomarkers are the most eective for predicting the biological age of worms will be helpful for the *C. elegans* aging research eld.

Recent studies have also demonstrated changes in the levels of proteins and metabolites as well as RNAs during aging. As aging is a stochastic process that inuences many dierent biological components, the analysis of these factors globally using omics technologies will be crucial for the progress of the aging research eld. Moreover, the integration of omics data with physiological and molecular markers of aging will provide even more powerful tools toward a better understanding of the mysteries of organismal aging.

## CONFLICT OF INTEREST

None declared.
